# Anatomical variants of the intercostobrachial nerve and its preservation during surgery, a systematic review and meta-analysis

**DOI:** 10.1186/s12957-024-03374-w

**Published:** 2024-04-11

**Authors:** Roberto Cirocchi, Matteo Matteucci, Justus Randolph, Francesca Duro, Luca Properzi, Stefano Avenia, Bruno Amato, Ruggiero Iandoli, Giovanni Tebala, Carlo Boselli, Piero Covarelli, Paolo Sapienza

**Affiliations:** 1 Department of Medicine and Surgery, University of Perugia, Perugia, 06132 Italy; 2 Department of Medicine and Surgery, University of Milan, Milan, 20122 Italy; 3 Georgia Baptist College of Nursing, Mercer University, Atlanta, GA 30341 USA; 4 Department of Public Health, University of Naples “Federico II”, Naples, 80131 Italy; 5 Department of General Surgery, P.O Frangipane Ariano Irpino, Avellino, 83031 Italy; 6 Department of Digestive and Emergency Surgery, AOSP of Terni, Terni, 05100 Italy; 7 Department of Surgery, “Sapienza“ University of Rome, Roma, 00161 Italy

## Abstract

**Background:**

The anatomic variants of the intercostobrachial nerve (ICBN) represent a potential risk of injuries during surgical procedure such as axillary lymph node dissection and sentinel lymph node biopsy in breast cancer and melanoma patients. The aim of this systematic review and meta-analysis was to investigate the different origins and branching patterns of the intercostobrachial nerve also providing an analysis of the prevalence, through the analysis of the literature available up to September 2023.

**Materials and methods:**

The protocol for this study was registered on PROSPERO (ID: CRD42023447932), an international prospective database for reviews. The PRISMA guideline was respected throughout the meta-analysis. A systematic literature search was performed using PubMed, Scopus and Web of Science. A search was performed in grey literature through google.

**Results:**

We included a total of 23 articles (1,883 patients). The prevalence of the ICBN in the axillae was 98.94%. No significant differences in prevalence were observed during the analysis of geographic subgroups or by study type (cadaveric dissections and in intraoperative dissections). Only five studies of the 23 studies reported prevalence of less than 100%. Overall, the PPE was 99.2% with 95% Cis of 98.5% and 99.7%. As expected from the near constant variance estimates, the heterogeneity was low, I^2^ = 44.3% (95% CI 8.9%−65.9%), *Q* = 39.48, *p* = .012. When disaggregated by evaluation type, the difference in PPEs between evaluation types was negligible. For cadaveric dissection, the PPE was 99.7% (95% CI 99.1%–100.0%) compared to 99.0% (95% CI 98.1%–99.7%).

**Conclusions:**

The prevalence of ICBN variants was very high. The dissection of the ICBN during axillary lymph-node harvesting, increases the risk of sensory disturbance. The preservation of the ICBN does not modify the oncological radicality in axillary dissection for patients with cutaneous metastatic melanoma or breast cancer. Therefore, we recommend to operate on these patients in high volume center to reduce post-procedural pain and paresthesia associated with a lack of ICBN variants recognition.

**Supplementary Information:**

The online version contains supplementary material available at 10.1186/s12957-024-03374-w.

## Introduction

The Intercostobrachial nerve (ICBN) emerges from the second intercostal space and it traverses the axilla horizontally [1, 2]. Then it perforates the deep fascia of the arm, providing the sensory supply to the upper medial region of the arm [3]. The anatomy of ICBN represents a potential risk of unintentional injuries during routine surgical procedures such as axillary lymph node dissection (ALND) and sentinel lymph node biopsy (SLNB) [4].

Breast cancer stands as the most prevalent malignant in women throughout the world, affecting almost 12% of women in Western countries [5]. The ICBN seems to be frequently damaged during mastectomy procedures and other techniques such as ALND (Axillary Lymph Node Dissection) and SLNB (Sentinel Lymph Node Biopsy); some studies suggest that injuring ICBN could play a role in Persistent Pain After Breast Cancer Treatment (PPBCT) and reduction of sensory function in the affected area [1, 3]. Additionally, the possibility of injury of the ICBN should also be considered in axillary surgery for melanoma [6].

Researchers have demonstrated that ICBN preservation significantly reduce post-procedural paresthesia and improve quality of life after the treatment for breast cancer and axillary surgery for melanoma. In fact, damaging ICBN or one of its primary branches could represents a cause of dysesthesia, paresthesia and chronic pain in these patients. Surgeons need to be accurate during the exploration of the axillary region, as the initial divisions and the connections between the ICBN and the brachial plexus may be damaged [3]. The configuration of the ICBN has an important variability, showing multiple points of origin, patterns of division, and connections with other branches. If surgeons could know the real frequency, the characteristics of these division and the origin models of ICBN they could reduce the ICBN lesions and the post operatory morbility [3, 4].

The aims of this systematic review and meta-analysis are to assess the prevalence, the different origins and branching patterns of the intercostobrachial nerve, analyzing the literature available up to the present day.

## Materials and methods

### Search strategy

The protocol for this study was registered on PROSPERO (ID: CRD42023447932), an international prospective database for reviews.

The PRISMA (preferred reporting items for systematic reviews and meta-analyses) guideline was respected throughout the meta-analysis [7]. The identification of articles to be included in the meta-analysis was carried out with searches up to September 2023 in the following databases: PubMed, Scopus and Web of Science. The search strategy performed in PubMed is presented as follows: nerve and Intercostobrachial or nervus intercostobrachialis. No date or language restrictions were applied. Identification of additional studies eligible for the meta-analysis was performed by searching the references of all included articles.

### Evaluation of inclusion criteria

Eligibility for inclusion in this systematic review and meta-analysiss was performed by M.M. and R.C. All intraoperative or cadaveric studies reporting extractable prevalence data on the origin or branching of ICBN were included. Exclusion criteria included: case reports, case series, letters to the editor, or conference abstracts.

### Data extraction

Data from the included studies were extracted by R.C. and M.M. For each article, the following information have been extracted: first author and year of publication, nation of study, sample size (number of patients and number of axillae analyzed), time of enrollment, type of study, type of evaluation (cadaveric or surgical), type of surgical treatment, prevalence of ICBN, origin of ICBN, mode of branching of ICBN. All selected full text articles were evaluated by quality assessment and analysis of the risk of bias using the Anatomical Quality Assurance (AQUA) [Henry, B.M. et al. 2017 [8]].

## Results

### Study identification

**Fig. 1:**
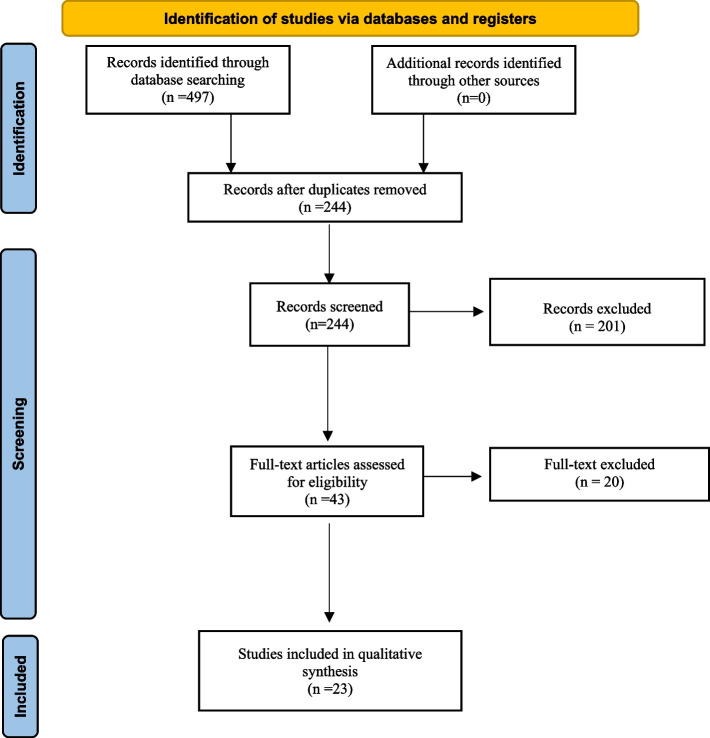
Prisma flow diagram of studies included

The initial literature search identified 497 articles. Following the removal of duplicates and primary screening, 43 articles were assessed as full text for eligibility in the meta-analysis records. Finally, we included a total of 23 articles [2–4, 6, 9–27] selected by eliminating articles with incomplete information and articles that used different classification (Fig. 1).

### Characteristics of the included studies

The systematic review and meta-analysis include twenty-three studies published between 1999 and 2023. Six studies were conducted on cadaveric samples [9, 15, 16, 23, 27, 28], and seventeen were performed intraoperatively [2–4, 6, 10–14, 17–22, 24–26]. The studies exhibit a broad geographic distribution, with 10 studies from Asia [3, 4, 9–12, 16, 18, 21, 28], 6 from Europe [6, 14, 19, 20, 25, 26], 4 from South America [2, 15, 22, 24], and one each from North America [23], Africa [17], and Australia [27]. Among the twenty-three studies, 1,636 patients were included, and 1,883 axillae were evaluated (494 from cadaveric dissections and 1,389 from intraoperative dissections): 765 from Asians, 570 from Europeans, 318 from South Americans, 200 from North Americans, 30 from Africans, and 28 from Australians (Table 1).

**Table 1:** Characteristics of the included studies

Author/Year	Nation	Time of enrollment	Type of study	Type of evaluation	Type of surgical treatment	Number of patients evaluated	Number of half-bodies
Özsahin/2023 [9]	Turkey	/	/	Cadaveric	/	8	16
Melhem/2021 [10]	Jordan	2018–2019	RCT	Surgery	ANDBC	48	48
Kaur/2020 [11]	India	NR	RCT	Surgery	ANDBC	53	53
Chirappapha/2019 [12]	Thiland	2016–2017	RCT	Surgery	ANDBC	43	43
Nayak/2018 [28]	India	/	/	Cadaveric	/	65	130
Orsolya/2017 [14]	Romania	2013–2015	OR	Surgery	ANDBC	100	100
Foroni/2017 [15]	Brasile	2010–2011	/	Cadaveric	/	30	60
Kailash/2016 [16]	India	NR	/	Cadaveric	/	30	60
Kumar/2016 [3]	India	2010–2012	RCT	Surgery	ANDBC	50	50
Darwish/2015 [17]	Egypt	2013–2014	OP	Surgery	ANDBC	30	30
Taira/2014 [18]	Japan	1998–2003	OP	Surgery	ANDBC	140	140
Andersen/2014 [19]	Denmark	2011–2013	OP	Surgery	ANDBC	133	133
Zhu/2014 [4]	2009–2010	OR	Surgery	ANDBC	156	156	
Soares/2014 [2]	Brasile	2012–2013	P	Surgery	ANDBC	100	100
Kubala/2013 [6]	Czech Republic	2007–2011	OP	Surgery	ANDM	113	113
Khan/2012 [20]	UK	NR	OP	Surgery	ANDBC	73	73
Verma/2009 [21]	India	2007–2009	R	Surgery	ANDBC	69	69
Ferreira/2008 [22]	Brasile	2005–2006	P	Surgery	ANDBC	73	73
Loukas/2006 [23]	USA	/	/	Cadaveric	/	100	200
Torresan/2003 [24]	Brasile	1999–2000	RCT	Surgery	ANDBC	85	85
Freeman/2002 [25]	UK	/	P	Surgery	ANDBC	73	73
Cunnick/2001 [26]	UK	1997–1998	P	Surgery	ANDBC	50	50
O’Rourke/1999 [27]	Australia	/	/	Cadaveric	/	14	28

*NR* Not Rated, *RCT* Randomized Control Trial, *OR* Observational Retrospective, *OP* Observational Prospective, *P* Prospective, *R* Retrospective, *ANDBC* Axillary Node Dissection performed during surgery for Breast Cancer

### Quality valutation of the studies included

**Fig. 2:**
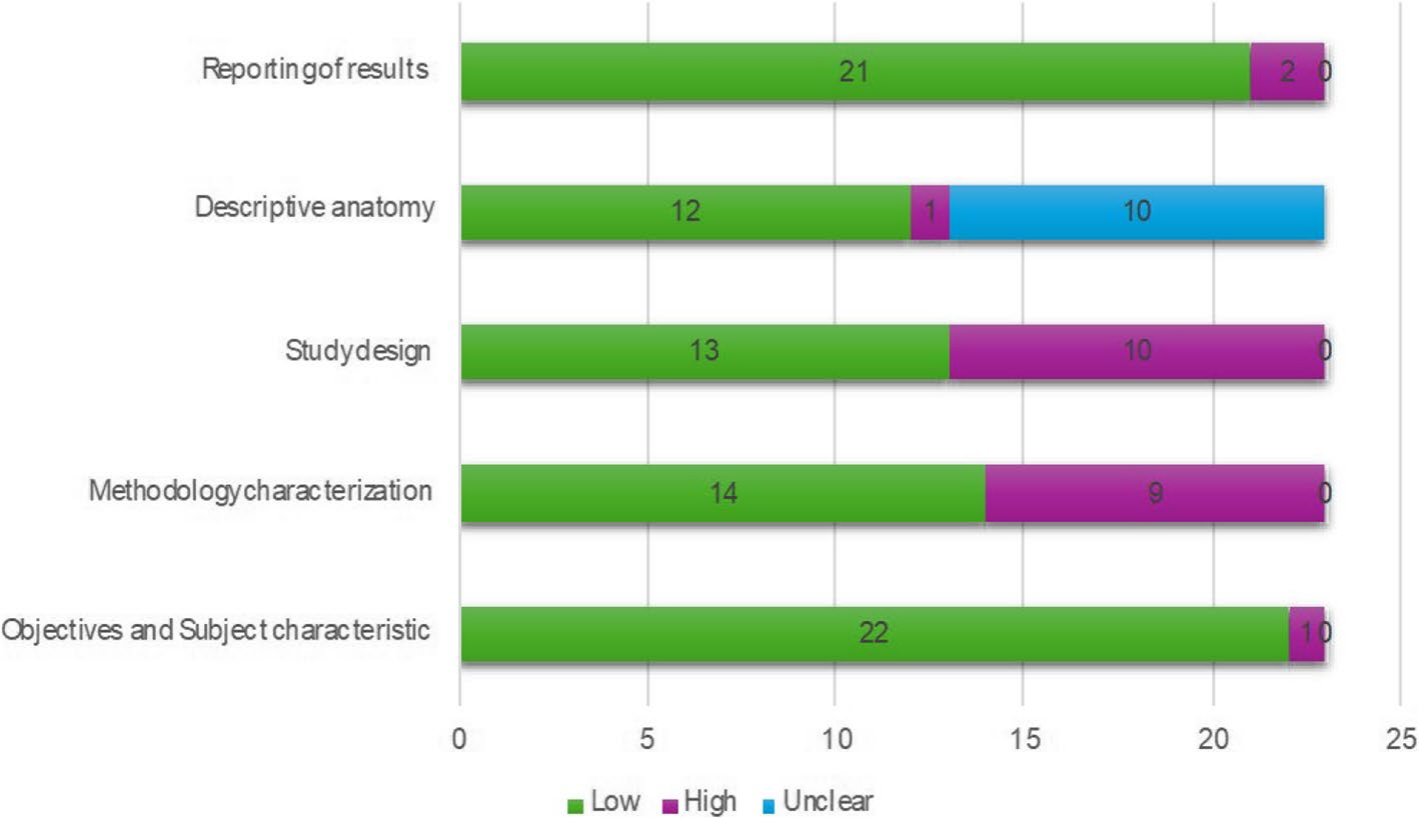
Assessment of quality and risk of bias by the AQUA tool

The AQUA tool probes for potential risk of bias in 5 studies domains (objectives and subject characteristics, study design, methodology characterization, descriptive anatomy and reporting of results) (Henry, B.M. et al. 2017 [8]). The risk of bias within each domain is normally categorized as “Low”, “High” or “Unclear”. Twenty-two of the studies included showed low risk in domain one (Objectives and Subject characteristics), ten studies showed high risk of bias in domain three (Methodology characterization), mainly because there is an important reduction of possibility of studying anatomy during an intervention. A summary of the assessment of quality and risk of bias by the AQUA tool is displayed in the Fig. 2.

### Statistical methods

For the primary outcome—prevalence of the ICBN—pooled prevalence estimates (PPES) and their 95% confidence intervals are reported using MetaXL software (V. 5.3). We used a DerSimonian and Larid random effects model with a double arcsin transformation, normalized prevalence, and a 0.5 continuity correction. Heterogeneity was investigated through the I^2^ statistics, Cochrane’s *Q* statistic, and a visual analysis of forest plots and funnel plots. In addition, we examined evaluation type (cadaveric dissection or intraoperative dissection) and geographic region of the first author’s affiliation (Africa, Americas, Asia, Oceania, or Europe) as factors. A leave-one-out sensitivity analysis was conducted to examine the effect of outlying studies. Funnel plots and Doi plots were used to investigate possible sources of bias—including publication bias. The leave-one-out sensitively analyses yielded PPEs from 99.3% with the Andersen 2014 [19] study excluded to 99.1% with the Loukas et al. 2016 [23] study excluded.

There was great variety in the outcome categories that authors used in the secondary outcomes: this is the reason why a single multicategory pooled prevalence estimate was not possible for any secondary outcomes. Furthermore, it was not feasible to report on the PPEs for tens of secondary outcomes if each of the outcome categories were reported as individual binary outcomes. Therefore, we descriptively report the secondary outcomes using only raw, marginal proportions.

## Results

### Primary outcome

#### Prevalence of the ICBN

**Fig. 3:**
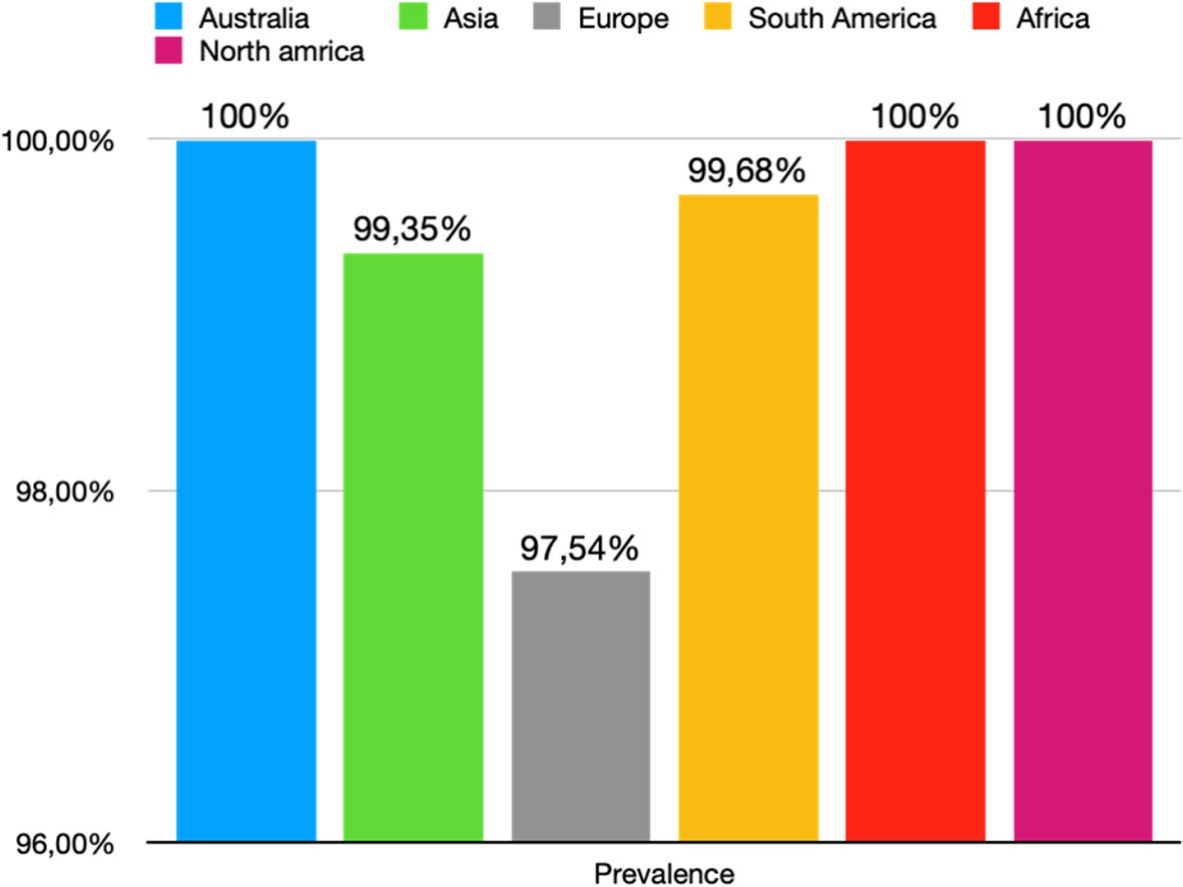
Prevalence of ICBN for continent

All studies reported data on the prevalence of the ICBN (1,883 axillae). The overall total prevalence of the ICBN in the axillae was 98.94% (1,863 ICBN). No significant differences in prevalence were observed during the analysis of geographic subgroups [99.35% in Asians (760 ICBN), 97.54% in Europeans (556 ICBN), 99.68% in South Americans (317 IBCN), 100% in North Americans (200 IBCN), 100% in Africans (30 ICBN), and 100% in Australians (28 IBCN)] or by study type [99.8% (93.75–100%) in cadaveric dissections (493 ICBN) and 98.63% (94.69–100%) in intraoperative dissections (1,370 ICBN)] (Fig. 3).

**Fig. 4:**
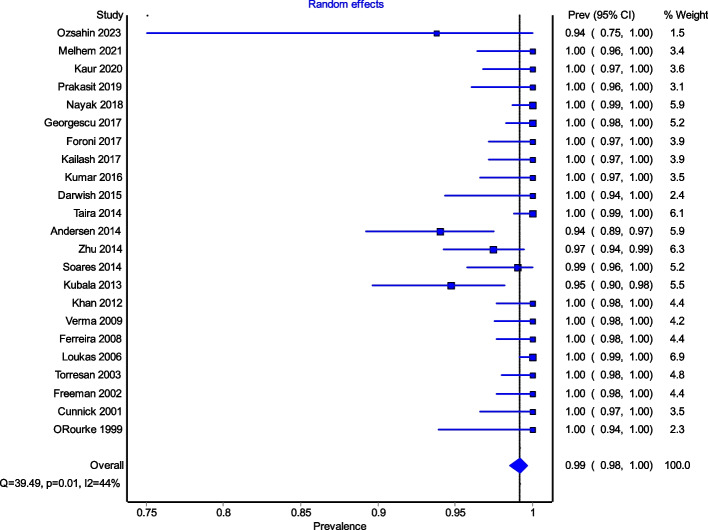
Prevalence estimates for the ICBN

**Fig. 5:**
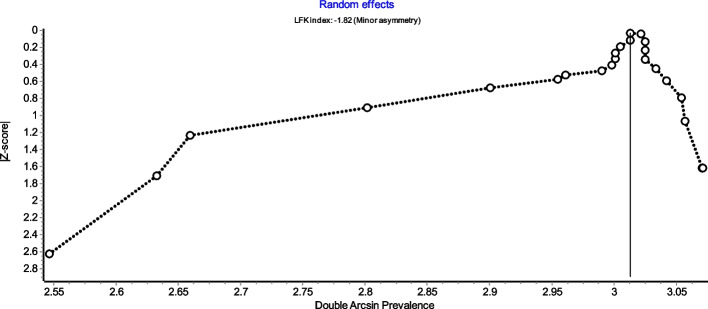
Doi plot

**Fig. 6:**
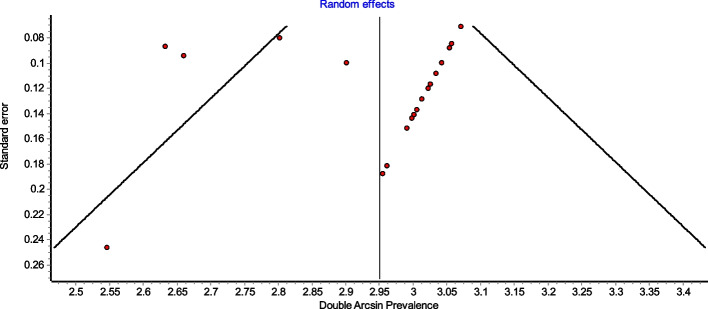
Funnel plot

**Fig. 7:**
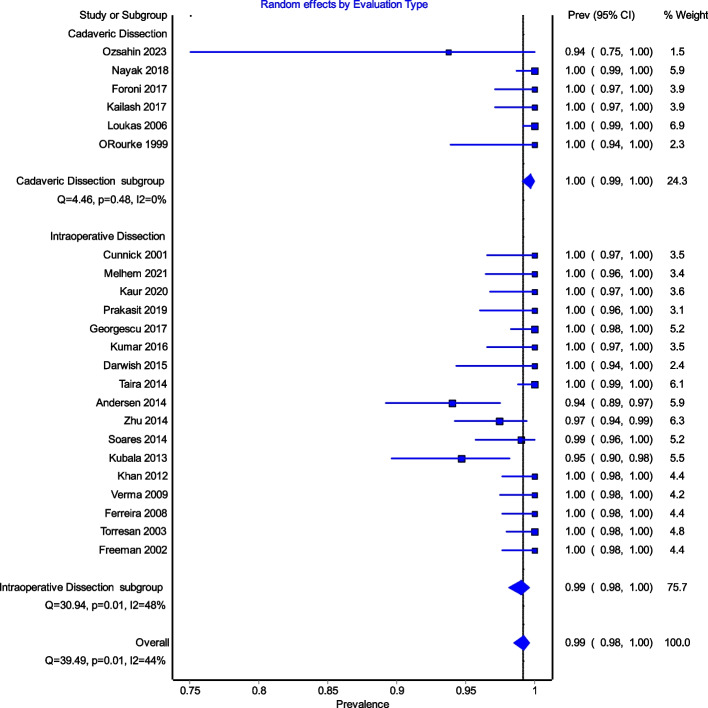
Random effects by Evaluation type

**Fig. 8:**
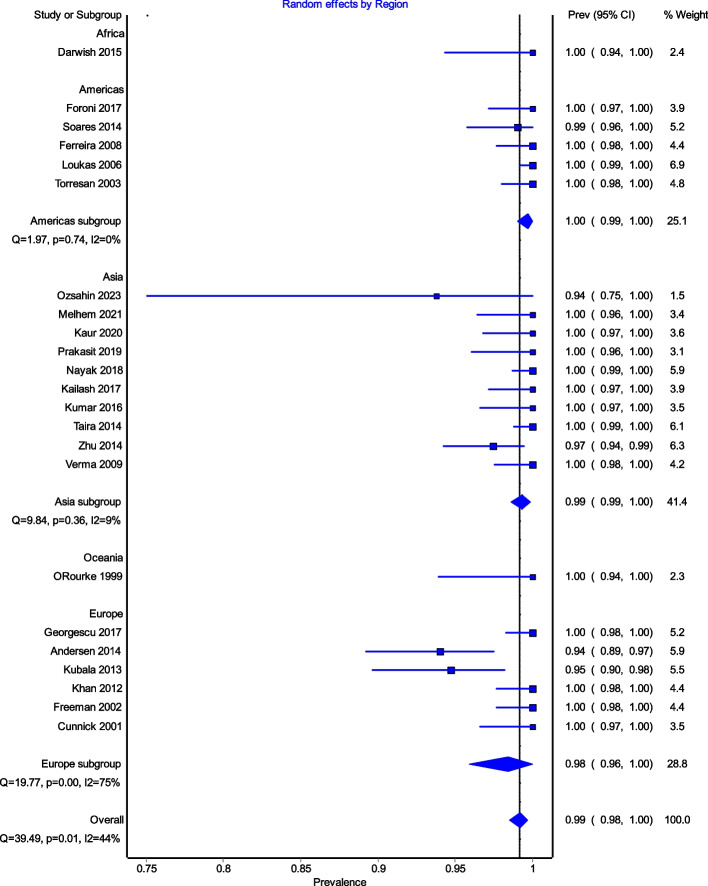
Random effects by Region

Figure 4 is a forest plot shows that the prevalence estimates for the ICBN had a near constant variance of 100%. Only five studies of the 23 studies reported prevalences of less than 100%. Overall, the PPE was 99.2% with 95% CIs of 98.5% and 99.7%. As expected from the near constant variance estimates, the heterogeneity was low, I^2^ = 44.3% (95% CI 8.9%–65.9%), *Q* = 39.48, *p* = .012. The Doi plot showed minor asymmetry (Fig. 5) and the funnel plot (Fig. 6) as indicative of an outcome with near constant variance. Rather than a random display of data points within a funnel pattern, there was a line of studies with a prevalence of 100% and the other data points to the left of that line. When disaggregated by evaluation type, the difference in PPEs between evaluation types was negligible (Fig. 7). For cadaveric dissection, the PPE was 99.7% (95% CI 99.1%–100.0%) compared to 99.0% (95% CI 98.1%–99.7%). There were differences in PPEs between subgroups was also negligible for geographic region (Fig. 8).: Africa = 99.2% (95% CI 94.3%–100.0%), Americas = 99.7% (95% CI 99.0%–100.0%), Asia = 99.3% (95% CI 98.6%–99.9%), Oceania = 99.2% (95% CI 93.9%–100.0%), and Europe = 98.4% (95% CI 95.9%–100.0%).

### Secondary outcomes

#### Origin of the intercostobrachial nerve

Eight studies (747 ICBN) reported data on the origin of the ICBN. The most common origin was at the T2 vertebral level, accounting for 81.79% of cases (611 ICBN), followed by T2-T3 at 8.17% (61 ICBN), T3 at 4.55% (34 ICBN), and T1-T2 at 2.9% (24 ICBN); much rarer origins were T1 at 1.07% (8 ICBN) and T1, T2, T3 at 1.2% (9 ICBN). To further clarify, combined origins were indicated when two separate roots were observed to merge into a common ICBN (Table 2). A total of 12 studies (1,060 ICBNs) reported data on the ICBN branching pattern. Table 3 provides detailed information on the ramifications of the ICBN. The ICBN most appeared as a single trunk in 51.6% of cases (547 ICBN), followed by the bifurcation pattern at 29.71% (315 IBCN), and the multiple branch pattern at 14.81% (157 IBCN). Subgroup analysis did not reveal significant differences.

**Table 2:** Studies with reported data on the origin of the ICBN

**Nation**	**Type of evaluation**	**Number of ICBN**	**T1**	**T2**	**T1 & T2**	**T2, & T3**	**T3**	**T1,T2,T3**	
Nayak 2018 [28]	Asia	Cadaveric dissection	130	5	98	0	0	27	0
Kumar 2016 [3]	Asia	Intraoperative dissection	50	0	48	2	0	0	0
Zhu 2014 [4]	Asia	Intraoperative dissection	152	2	118	17	6	0	9
Khan 2012 [20]	Europe	Intraoperative dissection	73	0	66	0	5	2	0
Verma 2009 [21]	Asia	Intraoperative dissection	69	0	68	1	0	0	0
Loukas 2006 [23]	America	Cadaveric dissection	200	0	146	0	50	4	0
Cunnick 2001 [26]	Europe	Intaroperative dissection	50	0	41	4	0	0	0
O’Rourke 1999 [27]	Australia	Cadaveric dissection	28	1	26	0	0	1	0

**Table 3:** Studies wich reported data on the ICBN branching pattern

First author	Nation	Type of evaluation	Number of ICBN	Single Trunk	Unification of two branches into Single Trunk	Total Bifurcation	Multiple Branches
Özşahin 2023 [9]	Asia	Cadaveric dissection	15	13	0	1	1
Nayak 2018 [28]	Asia	Cadaveric dissection	130	98	0	24	8
Foroni 2017 [15]	America	Cadaveric dissection	60	29	0	23	8
Kailash 2017 [16]	Asia	Cadaveric dissection	60	30	2	28	0
Kumar 2016 [3]	Asia	Intraoperative dissection	50	20	5	22	3
Zhu 2014 [4]	Asia	Intraoperative dissection	152	120	0	23	9
Soares 2014 [2]	America	Intraoperative dissection	99	61	0	25	13
Kubala 2013 [6]	Europe	Intraoperative dissection	107	55	1	38	13
Khan 2012 [20]	Europe	Intraoperative dissection	73	54	4	13	2
Verma 2009 [21]	Asia	Intraoperative dissection	69	48	1	20	0
Loukas 2006 [23]	America	Cadaveric dissection	200	0	20	80	100
Cunnick 2001 [26]	Europe	Intaroperative dissection	50	19	8	18	0

### Surgical analysis

#### Intraoperative preservation of the intercostobrachial nerve

Sixteen studies (1,311 ICBN) reported data on the preservation of the ICBN during axillary dissection for breast cancer (15 studies) and melanoma (1 study). It was found that the ICBN was completely preserved in 63.39% (34-95.65%) (831 ICBN) and partially preserved in 4.27% (15–29%) (56 ICBN). Only three studies reported the reasons for ICBN division (52 ICBN): accidental injury (53.85%, 28 ICBN), necessity dissection due to nerve involvement in lymph node clusters (30.77%, 16 ICBN), necessity dissection due to the nerve hindering proper access to the axillary cavity (15.38%, 8 ICBN).

### Sensorial analysis

#### Pain in patients with intercostobrachial nerve section

**Fig. 9:**
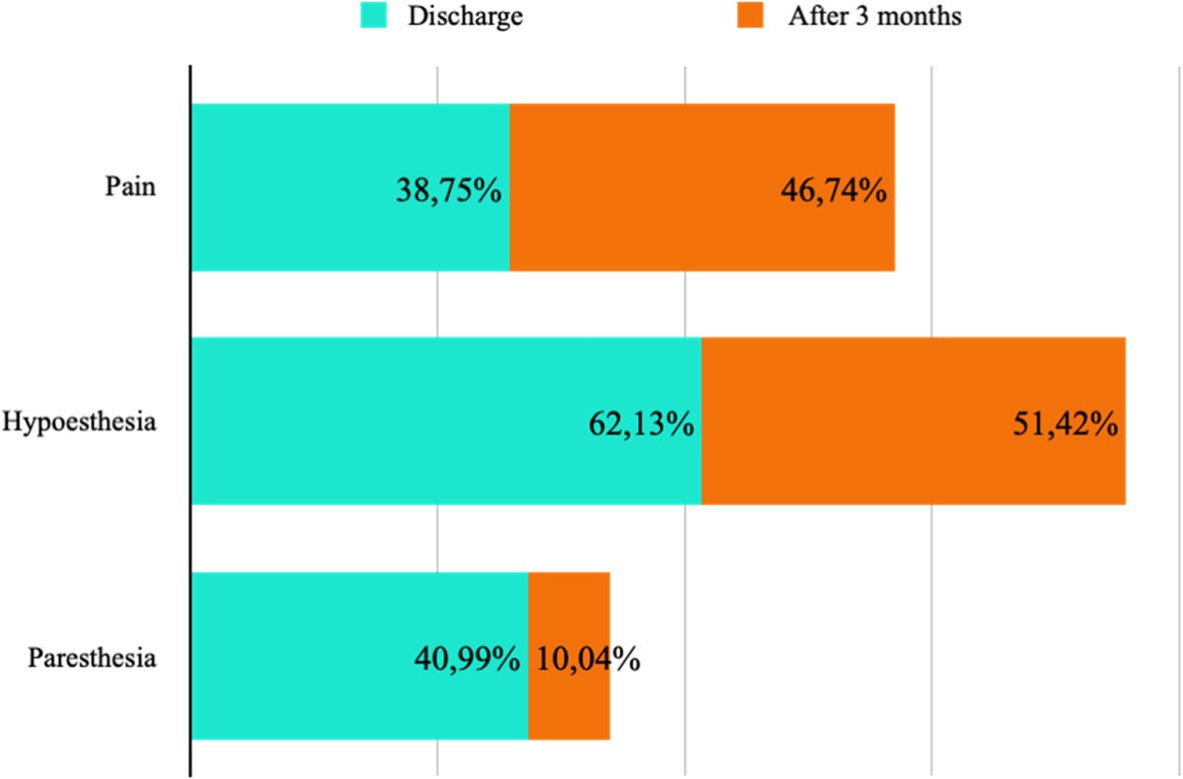
Patients with ICBN section

Postoperative pain was assessed at the time of discharge (7 studies, 209 ICBN) and after 3 months (4 studies, 92 ICBN). Postoperative pain was present in 38.75% at discharge and 46.74% after 3 months from the procedure (Fig. 9).

#### Hypoesthesia in patients with intercostobrachial nerve section

The decrease in postoperative sensitivity was evaluated at the time of discharge (5 studies, 169 ICBN) and after 3 months (4 studies, 105 ICBN). Postoperative hypoesthesia was present in 62.13% at the time of discharge and 51.42% after 3 months from the procedure (Fig. 9).

#### Paresthesia in patients with intercostobrachial nerve section

The presence of postoperative paresthesia was assessed at the time of discharge (9 studies, 283 ICBN) and after 3 months (2 studies, 42 ICBN). Postoperative paresthesia was present in 40.99% at the time of discharge and 10.04% after 3 months from the procedure (Fig. 9).

#### Pain in patients with intact intercostobrachial nerve

**Fig. 10:**
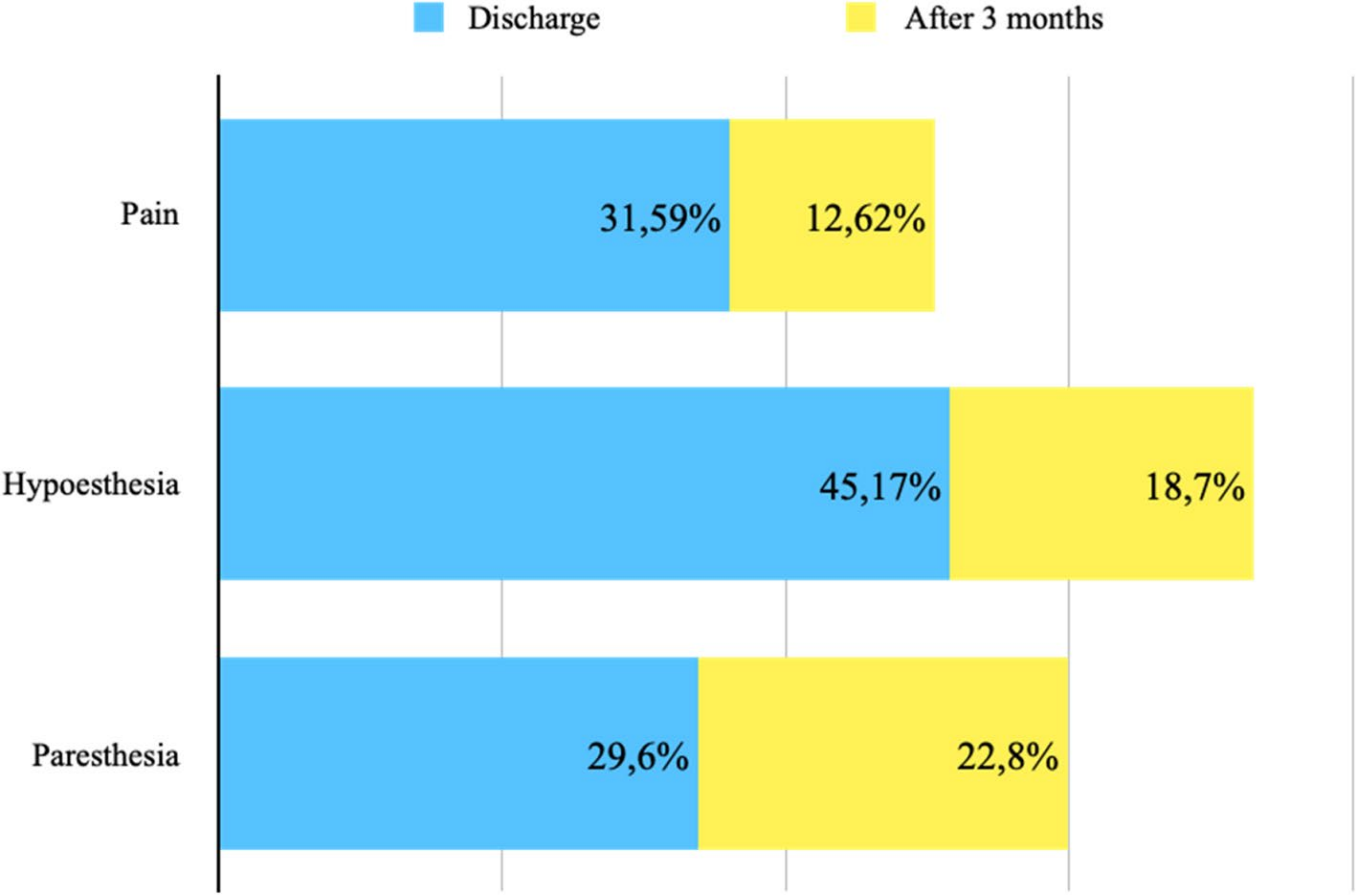
Patients with ICBN preservation

Postoperative pain was assessed at the time of discharge (7 studies, 288 ICBN) and after 3 months (4 studies, 103 ICBN). Postoperative pain was present in 31.59% at discharge and 12.62% after 3 months (Fig. 10).

#### Hypoesthesia in patients with intact intercostobrachial nerve

The decrease in postoperative sensitivity was evaluated at the time of discharge (5 studies, 228 ICBN) and after 3 months (4 studies, 123 ICBN). Postoperative hypoesthesia was present in 45.17% at discharge and 18.70% after 3 months (Fig. 10).

#### Paresthesia in patients with intact intercostobrachial nerve

The presence of postoperative paresthesia was assessed at the time of discharge (8 studies, 321 ICBN) and after 3 months (3 studies, 57 ICBN). Postoperative paresthesia was present in 29.6% at discharge and 22.8% after 3 months (Fig. 10).

## Discussion

The intercostobrachial nerve (ICBN) is an anatomical structure with a high variability in origin and branching pattern. The ICBN is a branch of the second intercostal nerve: therefore, special attention should be paid to the area of the second intercostal space, where the origin of the ICBN is most likely (90.6%). However, the ICBN could present occasional contribution also from the third intercostal nerve and it can be identified in an anterior position during the exposure of the thoracic and thoracodorsal long nerves. The ICBN is at high risk of injury during operative procedures into the axilla. ICBN fiber’s injury has been associated with post-procedural pain, paresthesia and with a reduction of the quality of life.

The evaluation of prevalence of ICBN might have an impact on surgical plan and patient outcomes: in fact, lymphedema, motor and/or sensor alterations of the arm have an impact on the quality of life of patients who underwent to operative procedure into the axillary region. Moderate or severe post-operative pain is experienced by 50% of patients after breast surgery (Andersen; Besic).

The ICBN should always be preserved. However, current guidelines do not provide specific recommendations regarding the preservation or sacrifice of the ICBN during axillary lymph-node dissection or sentinel lymph-node biopsy. A study conducted by Henry et al. in 2017 [29] showed that the ICBN is a variable structure at risk for injury during operative procedures of the axilla and due to the postoperative pain and paresthesia experienced by patients following injury, surgeons need to exercise caution and need to preserve the ICBN. Another study, conducted by Warrier et al. in 2014 [30] showed that the incidence of sensory disorder was lower in case of preservation of ICBN compared with the division of the ICBN.

On the other hand, in three different studies, conducted respectively by Abdullah et al. 1998 [31], Salmon et al. 1998 [32] and Torresan et al. 2003 [24], showed that patients who presented sensory distur in the immediate post-operative period may have a resolution of the symptoms and patients who may not notice any sensory disturbance initially may develop it at a later stage.

The ICBN can be damaged for a variety of reasons, from traction to transection. In addition, damaged nerves can develop neuromas that can further complicate the patient’ symptoms.

Our systematic review and meta-analysis showed that the overall prevalence of ICBN was 98.94% and that more frequently it exists as a single trunk (51.6%) originating from the T2 vertebral level (81.79%). The branching subgroups are differently based on the type of the study (cadaveric or operational). In fact, we noticed that the ICBNs examined in cadavers (99.8%) had a bifurcation rate more than double than those evaluated during operation (98.63%).

From these differences we deduced two different hypotheses. Firstly, the limited intraoperative field of view and the inability to freely dissect tissue without consequences may limit the in vivo identification of the nerve branches. These differences may help to explain the reason why post-operative complications might be present even if nerves are successfully identified and protected. Kumar et al. 2016 [3] noted that, six months after surgery, 20% of patients who had successfully preserved ICBN still had numbness and paresthesia. The ICBN may also be damaged due to stretching by retractors or other intraoperative stresses on nerve fibers. Secondly, ICBN might have a propensity to bifurcate unequally when analyzing cadaveric or intraoperative studies (63.4% unequal bifurcation versus 36.6% equal bifurcation). Therefore, surgeons can easily identify the larger trunk, but a failure to identify the smaller branch puts it at high risk of operative injury.

Our meta-analysis was limited by several factors related to inconsistent reporting and small sample sizes. Some studies presented the origins of the first or third intercostal nerve as separate data. It was unclear whether these origins were duplications or simply contributing fibers and therefore were excluded from the present analysis. In addition, we observed a high heterogeneity which might have the result of an inherent variable nature of the ICBN course. Another limitation of our study is the lack of data regarding the preservation of the intercostobrachial nerve during SLNB. However, larger studies are needed to demonstrate this observation.

Furthermore, in our analysis, cadaveric dissection rather than intraoperative dissection was more specific in the demonstration of multiple branching pattern. In fact, anatomical data retrieved from operative field were less accurate. Probably this rate discrepancy was associated to reporting errors.

In general, the studies did not provide information on factors such as sex and laterality, which could allow further subgroup analyses. Most studies have been conducted on women; however, Loukas et al. 2006 [23] found no gender differences. Limited data from regions such as Africa, North America, Oceania, and South America precluded analysis of other regions besides Europe and Asia. Many studies have a lack of detailed data on the branching site or symmetry of post-division branches.

Future studies need to be carried out to further elucidate the behavior of the terminal branching of the ICBN and its anastomoses with the brachial plexus. There is also the possibility of studying the use of landmarks or relationships with adjacent structures to be able to intraoperatively identify the nerves. Structures such as the lateral thoracic vein discussed in O’Rourke et al. 1999 [27] could be a potential candidate. Integration with future research will provide valuable information for surgeons and, ideally, lead to more positive outcomes for patients undergoing surgeries in the axillary region. In fact, ICBN neuralgia and post-operative pain syndrome can be successfully managed with loco-regional anesthesia techniques, but the primary goal should be the overall reduction of their incidence.

An accurate knowledge of the anatomy of axillary region is crucial to reduce nerve’ injuries. Another possible strategy for minimizing nerve’ injuries during surgery could be the ultrasound guided identification of ICBN before surgery. In fact, in a study conducted by Feigl et al. the ICBN blocks with ultrasound guided anterior approach to intercostal nerves was evaluated to supplement the axillary block. The sonographic identification of ICBN and its possible anatomical variations may be useful to reduce ICBN’s injuries [33].

Furthermore, the group with intact ICBN have a higher percentage of paresthesia (32.8%) after three months than the group with ICBN section. We were unable to explain this result, but we think that this pain might be similar to groin pain after inguinal hernia repair [34]. For this reason, some surgeons suggest to perform the inguinal nerve neurectomy in all patient underwent open mesh hernia repair [35].

## Conclusion

The prevalence of ICBN is very high, for this reason during axillary dissection a small operative theatre is associated at the inability to freely dissect tissue without a reduction of post-procedural pain and paresthesia. The division of the ICBN during axillary lymph node dissection, increases the risk of sensory disturbance such as hyposensitivity of the arm. The preservation of the ICBN do not modify the oncological radicality in axillary dissection for patients with cutaneous metastatic melanoma or breast cancer.

### Supplementary Information

**Supplementary Material 1.**

## Data Availability

The data of the article can be accessed by requesting the corresponding author.

## References

[1] Mewa Kinoo S, Singh B (2016). Complex regional pain syndrome of the breast and chest wall. Breast J.

[2] Soares EW (2014). Anatomical variations of the axilla. Springerplus.

[3] Kumar P, Meena RN, Sheikh BH, Belliappa V, Pais AV (2016). Intercostobrachial nerve - anatomical considerations and its importance in carcinoma breast of female patients. Ann Surg Perioper Care.

[4] Zhu JJ, Liu XF, Zhang PL, Yang JZ, Wang J, Qin Y, Zhang GL (2014). Anatomical information for intercostobrachial nerve preservation in axillary lymph node dissection for breast cancer. Genet Mol Res.

[5] DeSantis CE, Ma J, Gaudet MM, Newman LA, Miller KD, Goding Sauer A (2014). Breast cancer statistics, 2013. CA Cancer J Clin.

[6] Kubala O, Prokop J, Jelínek P, Ostruszka P, Tošenovský J, Ihnát P (2013). Anatomicko-chirurgická studie průběhu interkostobrachiálních nervů (ICBN) v axile při exenteraci I. a II. etáže axily u karcinomu prsu a maligního melanomu [Anatomic-surgical study of intercostobrachial nerve (ICBN) course in axilla during I. and II. Level of axilla clearance in breast cancer and malignant melanoma]. Rozhl Chir.

[7] Page MJ, McKenzie JE, Bossuyt PM (2021). The PRISMA 2020 statement: an updated guideline for reporting systematic reviews. BMJ.

[8] Henry BM, Tomaszewski KA, Ramakrishnan PK (2017). Development of the Anatomical Quality Assessment (AQUA) Tool for the quality assessment of anatomical studies included in meta-analyses and systematic reviews. Clin Anat.

[9] Özşahin MK, Kaynak G, Afacan MY (2023). Anatomical variations of intercostobrachial nerve: a potential candidate for neurotization after traumatic median nerve injury?. Ulus Travma Acil Cerrahi Derg.

[10] Melhem J, Amarin M, Odeh G, Al-Bustami N, Al-Lauzy H, Ayoub R (2021). Intercostobrachial nerve (ICBN) preservation versus sacrifice in axillary dissection: randomized controlled trial. Am J Clin Oncol.

[11] Kaur N, Kumar R, Jain A (2021). Sensory changes and postmastectomy pain following preservation of intercostobrachial nerve in breast cancer surgery: a prospective randomized study. Indian J Surg Oncol.

[12] Chirappapha P, Kongdan Y, Vassanasiri W, Ratchaworapong K, Sukarayothin T, Supsamutchai C (2019). Evaluation the effect of preserving intercostobrachial nerve in axillary dissection for breast cancer patient. Gland Surg.

[13] Soubhagya Ranjan Nayak, Smita Singh Banerjee (2019). Anatomic variations of the extrathoracic course of the intercostobrachial nerve and its clinical significance. Gland Surg.

[14] Orsolya HB, Coros MF, Stolnicu S, Georgescu R (2017). Does the surgical management of the intercostobrachial nerve influence the Postoperatory Paresthesia of the upper limb and life quality in breast cancer patients?. Chirurgia (Bucur)..

[15] Foroni L, Siqueira MG, Martins RS, Oliveira GP (2017). The intercostobrachial nerve as a sensory donor for hand reinnervation in brachial plexus reconstruction is a feasible technique and may be useful for restoring sensation. Arq Neuropsiquiatr.

[16] Kailash D, Balkund S, Priyadharshini (2017). A study on variations of intercostobrachial nerve. MedPulse – Int J Anat.

[17] Darwish A, Mlees A, El-Gendy M, Elghazeery M (2015). Preservation of lateral thoracic vein and intercostobrachial nerve in breast conservative surgery. Ain Shams J Surg.

[18] Taira N, Shimozuma K, Ohsumi S (2014). Impact of preservation of the intercostobrachial nerve during axillary dissection on sensory change and health-related quality of life 2 years after breast cancer surgery. Breast Cancer.

[19] Andersen KG, Aasvang EK, Kroman N, Kehlet H (2014). Intercostobrachial nerve handling and pain after axillary lymph node dissection for breast cancer. Acta Anaesthesiol Scand.

[20] Khan A, Chakravorty A, Gui GP (2012). In vivo study of the surgical anatomy of the axilla. Br J Surg.

[21] Verma S, Kala R, Bhargava G, Yadav R, Singh R, Maurya P (2009). Evaluation of the role of preservation of the intercostobrachial nerve on the post-mastectomy pain syndrome in breast cancer patients of North India. Internet J Surg 23.

[22] Ferreira BP, Soares. Morbidade cirúrgica pós-biópsia de linfonodo sentinela e esvaziamento axilar: estudo comparativo em mulheres com e sem preservação do nervo intercostobraquial. Rev Assoc Med Bras. 2008;54(6):517–21. 10.1590/s0104-42302008000600016 19197529

[23] Loukas M, Hullett J, Louis RG, Holdman S, Holdman D (2006). The gross anatomy of the extrathoracic course of the intercostobrachial nerve. Clin Anat.

[24] Torresan RZ, Cabello C, Conde DM, Brenelli HB (2003). Impact of the preservation of the intercostobrachial nerve in axillary lymphadenectomy due to breast cancer. Breast J.

[25] Freeman SR, Washington SJ, Pritchard T, Barr L, Baildam AD, Bundred NJ (2003). Long term results of a randomised prospective study of preservation of the intercostobrachial nerve. Eur J Surg Oncol.

[26] Cunnick GH, Upponi S, Wishart GC (2001). Anatomical variants of the intercostobrachial nerve encountered during axillary dissection. Breast.

[27] O’Rourke MG, Tang TS, Allison SI, Wood W (1999). The anatomy of the extrathoracic intercostobrachial nerve. Aust N Z J Surg.

[28] Nayak SR, Banerjee SS (2018). Anatomic variations of the extrathoracic course of the intercostobrachial nerve and its clinical significance. Asian J Med Sci.

[29] Henry BM, Graves MJ, Pękala JR, Sanna B, Hsieh WC, Tubbs RS (2017). Origin, branching, and communications of the intercostobrachial nerve: a meta-analysis with implications for Mastectomy and axillary lymph node dissection in breast cancer. Cureus.

[30] Warrier S, Hwang S, Koh CE, Shepherd H, Mak C, Carmalt H (2014). Preservation or division of the intercostobrachial nerve in axillary dissection for breast cancer: meta-analysis of randomised controlled trials. Breast.

[31] Abdullah TI, Iddon J, Barr L, Baildam AD, Bundred NJ (1998). Prospective randomized controlled trial of preservation of the intercostobrachial nerve during axillary node clearance for breast cancer. Br J Surg.

[32] Salmon RJ, Ansquer Y, Asselain B (1998). Preservation versus section of intercostal-brachial nerve (IBN) in axillary dissection for breast cancer–a prospective randomized trial. Eur J Surg Oncol.

[33] Feigl GC, Litz RJ, Marhofer P (2020). Anatomy of the brachial plexus and its implications for daily clinical practice: regional anesthesia is applied anatomy. Reg Anesth Pain Med..

[34] Charalambous MP, Charalambous CP (2018). Incidence of chronic groin pain following open mesh inguinal hernia repair, and effect of elective division of the ilioinguinal nerve: meta-analysis of randomized controlled trials. Hernia.

[35] Cirocchi R, Sutera M, Fedeli P, Anania G, Covarelli P, Suadoni F, Boselli C, Carlini L, Trastulli S, D’Andrea V, Bruzzone P (2021). Ilioinguinal nerve neurectomy is better than preservation in Lichtenstein Hernia repair: a systematic literature review and meta-analysis. World J Surg.

