# Supplementary material for: Anatomical variants of the intercostobrachial nerve and its preservation during surgery, a systematic review and meta-analysis
Source: World J Surg Oncol. 2024 Apr 11;22:92. doi: 10.1186/s12957-024-03374-w (PMC11007944; doi:10.1186/s12957-024-03374-w)
Supplement: Supplementary file 1 — Supplementary Material 1. [file 12957_2024_3374_MOESM1_ESM.docx]

**Identification of studies via databases and registers**

Records removed *before screening*:

Duplicate records removed (n =253)

Records marked as ineligible by automation tools (n = 0)

Records removed for other reasons (n = 0)

Records identified from*:

Databases (Medline/PubMed N = 191, Scopus N = 233, WOS N = 73)

**Identification**

Records screened

(n = 244)

Records excluded

(n = 201)

Reports sought for retrieval

(n = 43)

Reports not retrieved

(n = 0)

**Screening**

Reports excluded: (n = 20)

Reports assessed for eligibility

(n = 27)

Studies included in review

(n = 23)

**Included**

SDC1

*From:*  Page MJ, McKenzie JE, Bossuyt PM, Boutron I, Hoffmann TC, Mulrow CD, et al. The PRISMA 2020 statement: an updated guideline for reporting systematic reviews. BMJ 2021;372:n71. doi: 10.1136/bmj.n71

For more information, visit: <http://www.prisma-statement.org/>
